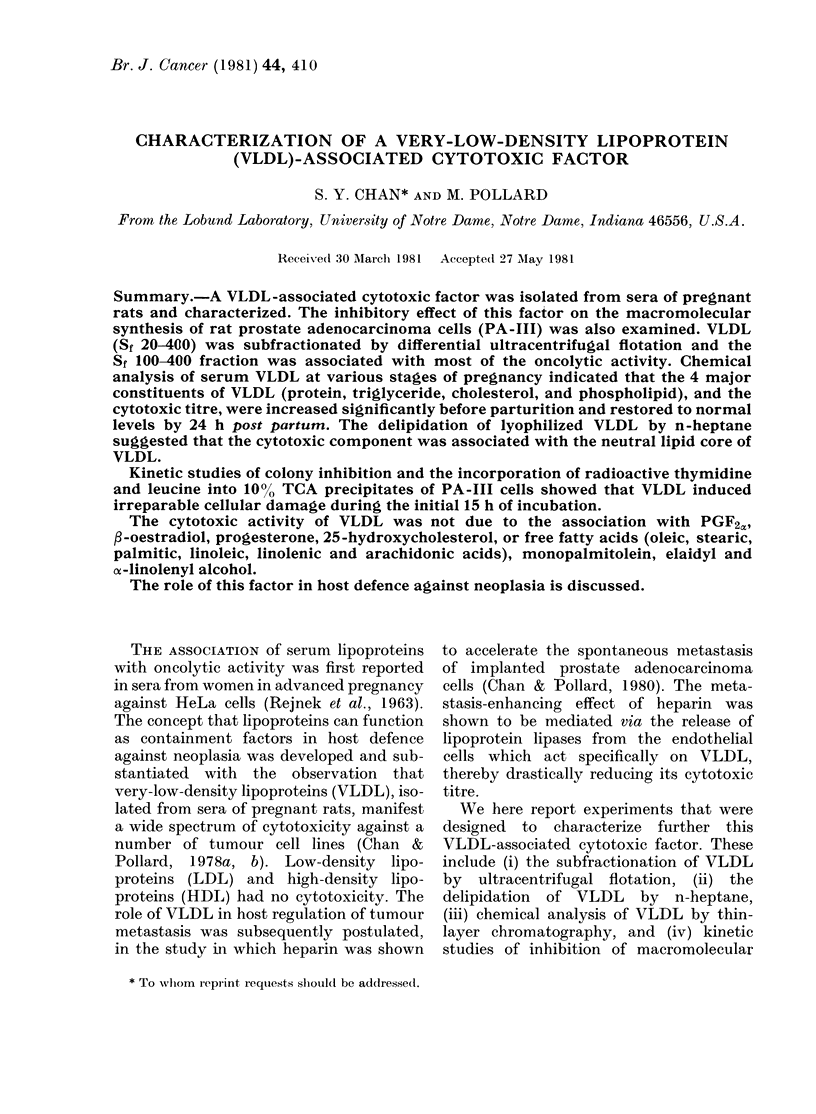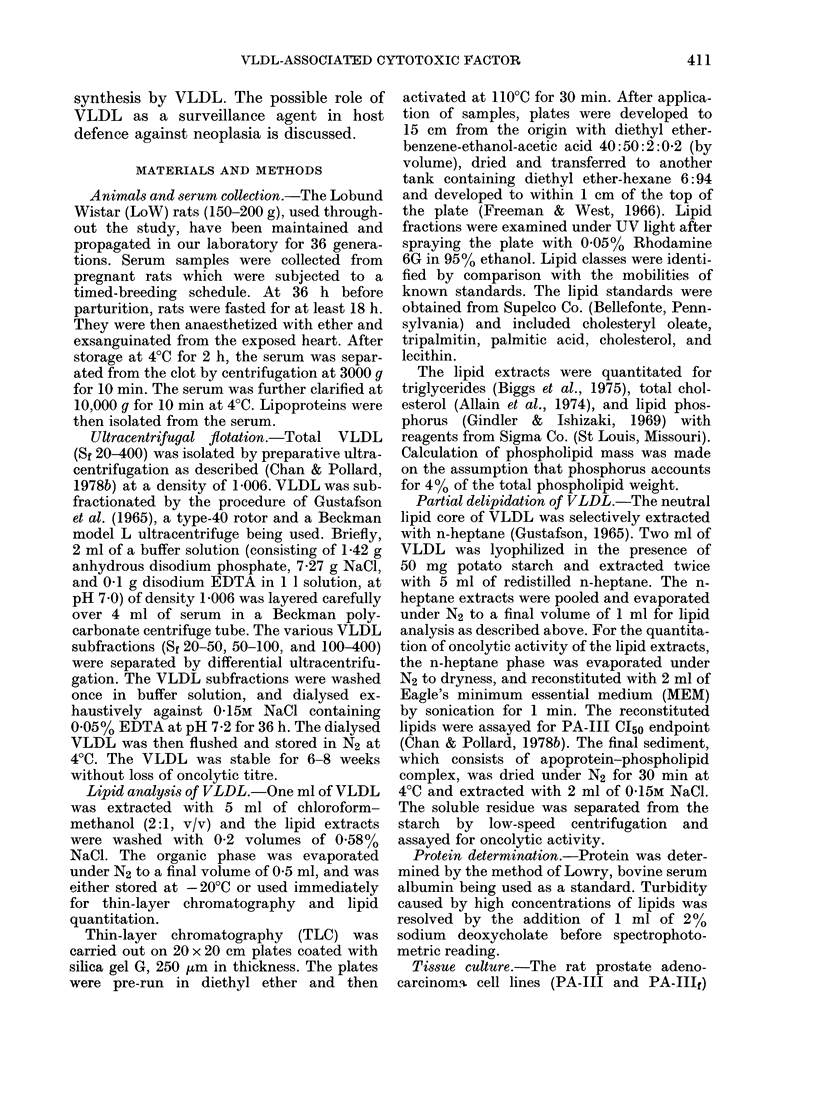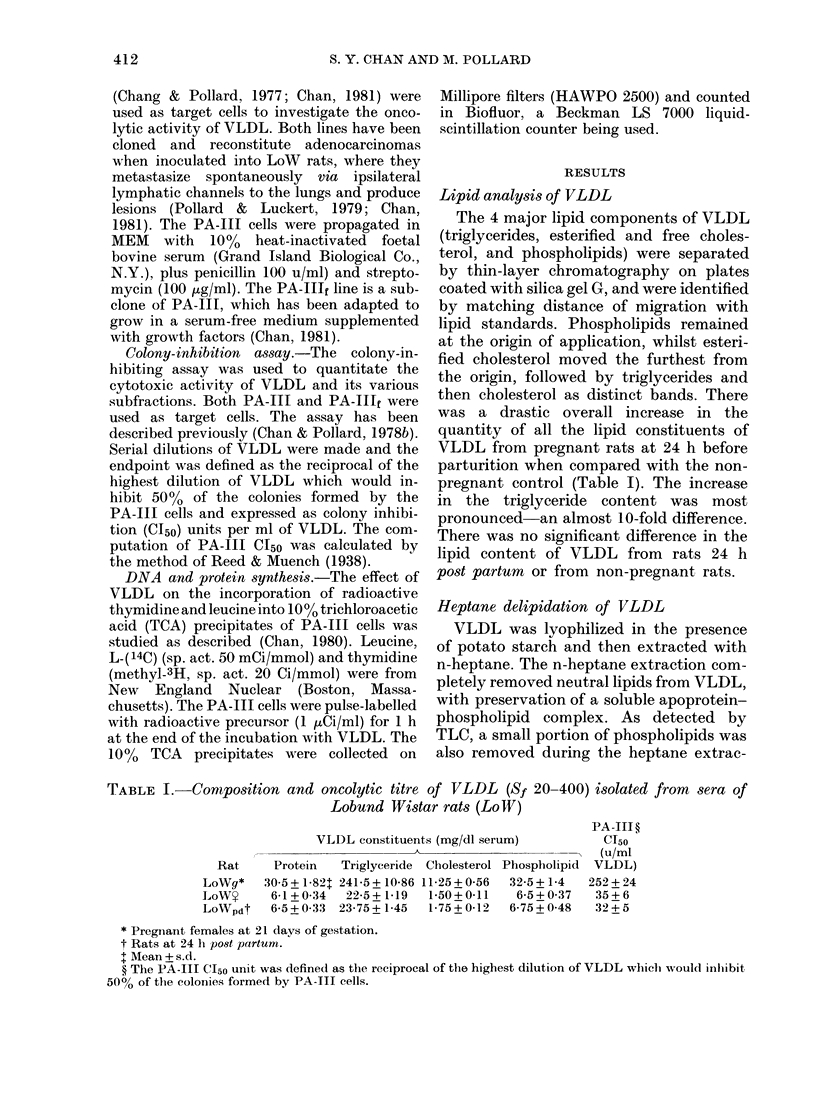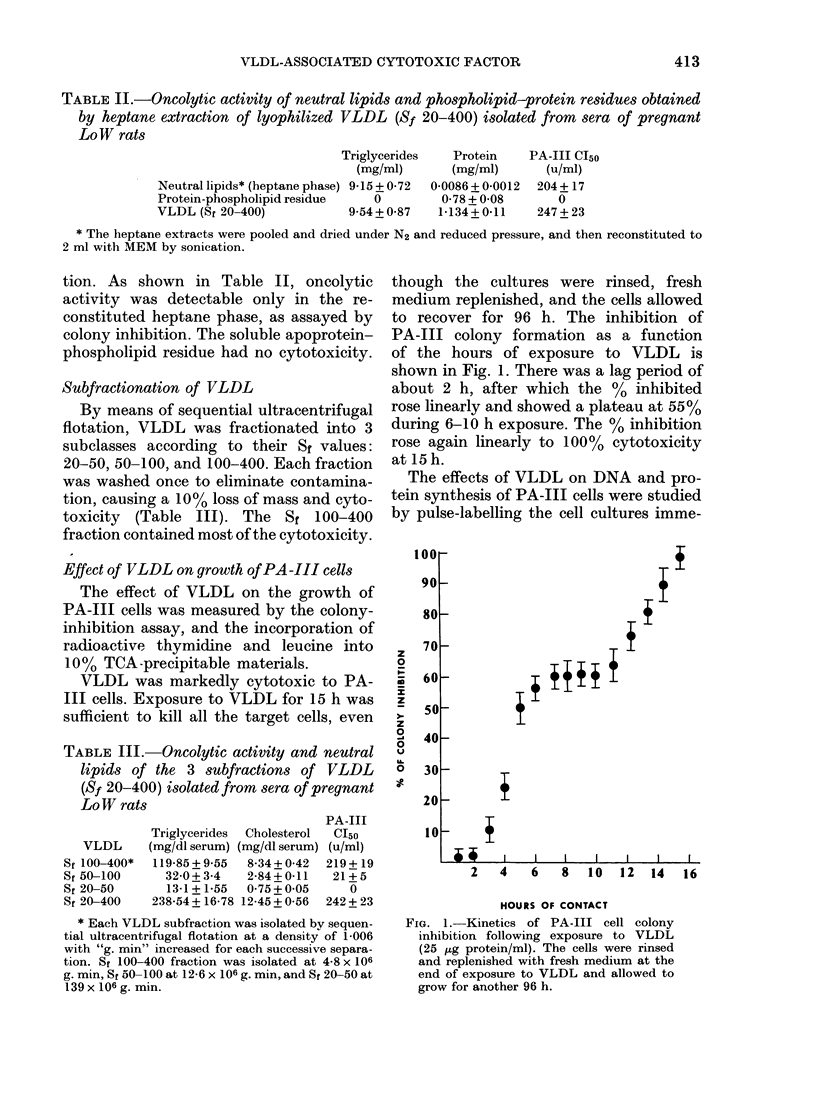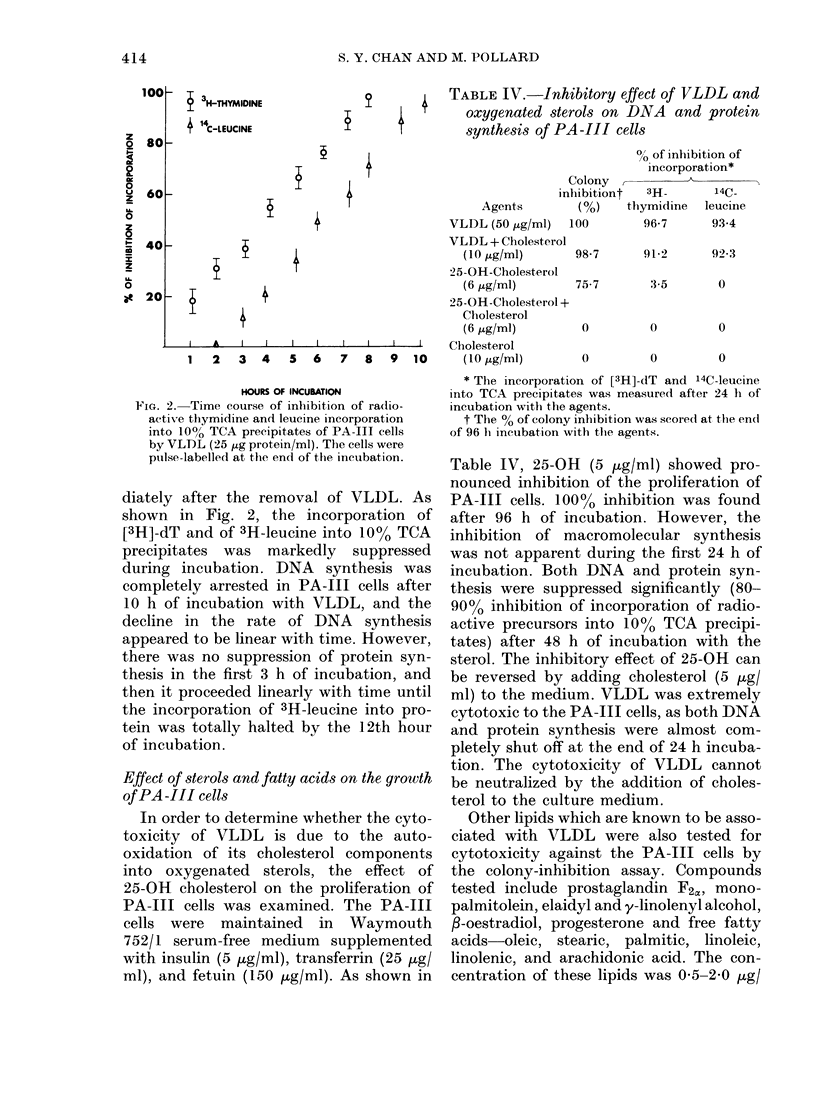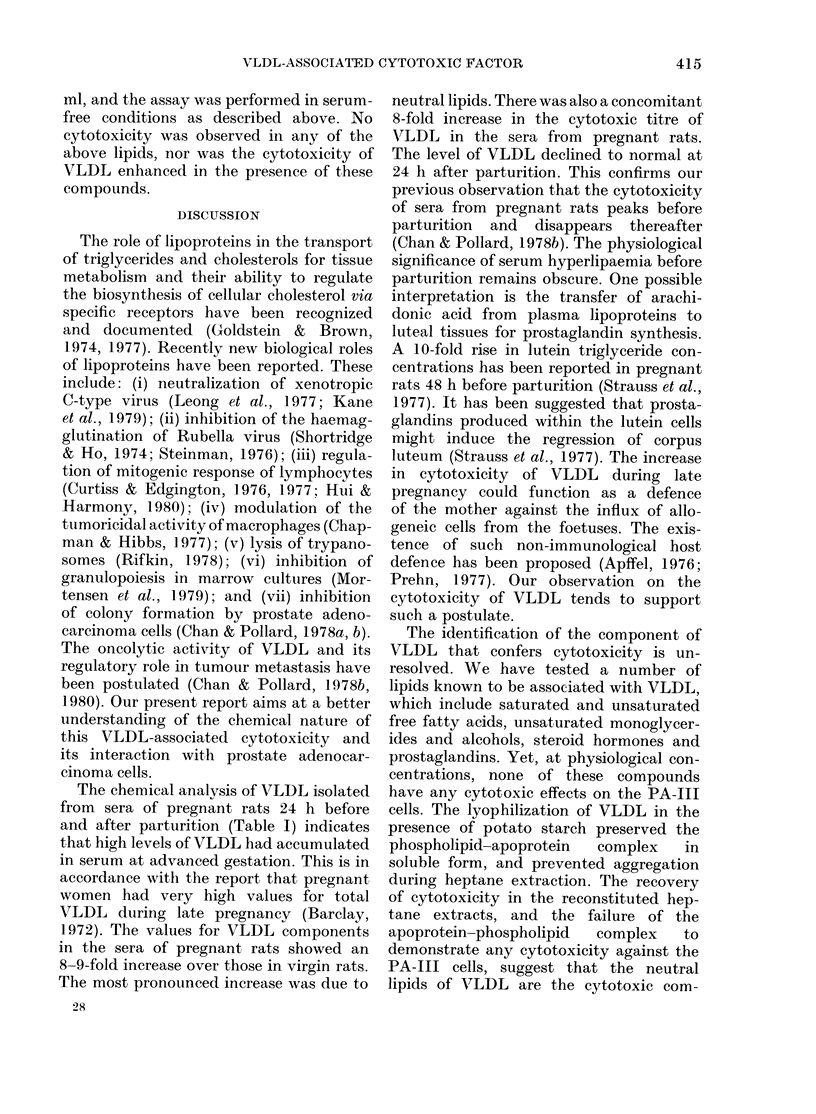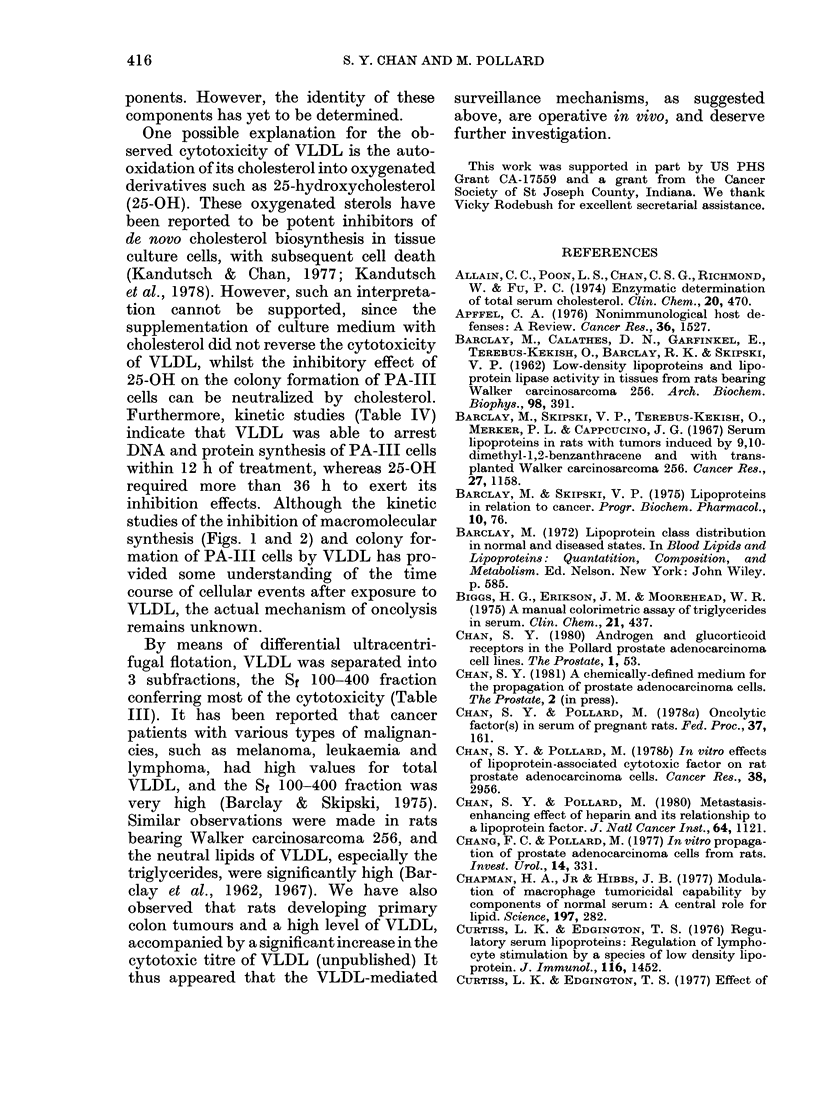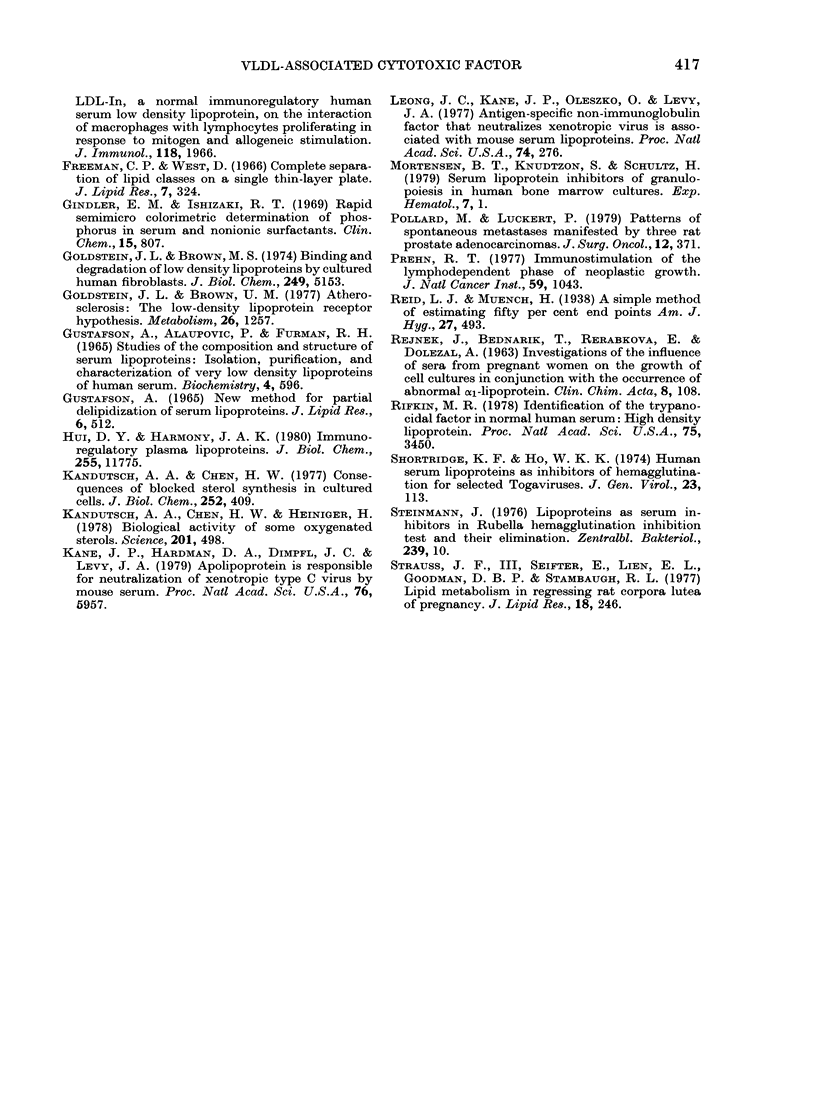# Characterization of a very-low-density lipoprotein (VLDL)-associated cytotoxic factor.

**DOI:** 10.1038/bjc.1981.199

**Published:** 1981-09

**Authors:** S. Y. Chan, M. Pollard

## Abstract

A VLDL-associated cytotoxic factor was isolated from sera of pregnant rats and characterized. The inhibitory effect of this factor on the macromolecular synthesis of rat prostate adenocarcinoma cells (PA-III) was also examined. VLDL (Sf 20-400) was subfractionated by differential ultracentrifugal flotation and the Sf 100-400 fraction was associated with most of the oncolytic activity. Chemical analysis of serum VLDL at various stages of pregnancy indicated that the 4 major constituents of VLDL (protein, triglyceride, cholesterol, and phospholipid), and the cytotoxic titre, were increased significantly before parturition and restored to normal levels by 24 h post partum. The delipidation of lyophilized VLDL by n-heptane suggested that the cytotoxic component was associated with the neutral lipid core of VLDL. Kinetic studies of colony inhibition and the incorporation of radioactive thymidine and leucine into 10% TCA precipitates of PA-III cells showed that VLDL induced irreparable cellular damage during the initial 15 h of incubation. The cytotoxic activity of VLDL was not due to the association with PGF2 alpha, beta-oestradiol, progesterone, 25-hydroxycholesterol, or free fatty acids (oleic, stearic, palmitic, linoleic, linolenic and arachidonic acids), monopalmitolein, elaidyl and alpha-linolenyl alcohol. The role of this factor in host defence against neoplasia is discussed.


*Br. J. Cancer* (1981) **44**, 410

### CHARACTERIZATION OF A VERY-LOW-DENSITY LIPOPROTEIN (VLDL)-ASSOCIATED CYTOTOXIC FACTOR

S. Y. CHAN* AND M. POLLARD

*From the Lobund Laboratory, University of Notre Dame, Notre Dame, Indiana* 46556, *U.S.A.*

Received 30 March 1981 Accepted 27 May 1981

**Summary.—A VLDL-associated cytotoxic factor was isolated from sera of pregnant rats and characterized. The inhibitory effect of this factor on the macromolecular synthesis of rat prostate adenocarcinoma cells (PA-III) was also examined. VLDL ($S_f$ 20–400) was subfractionated by differential ultracentrifugal flotation and the $S_f$ 100–400 fraction was associated with most of the oncolytic activity. Chemical analysis of serum VLDL at various stages of pregnancy indicated that the 4 major constituents of VLDL (protein, triglyceride, cholesterol, and phospholipid), and the cytotoxic titre, were increased significantly before parturition and restored to normal levels by 24 h *post partum*. The delipidation of lyophilized VLDL by n-heptane suggested that the cytotoxic component was associated with the neutral lipid core of VLDL.**

**Kinetic studies of colony inhibition and the incorporation of radioactive thymidine and leucine into 10% TCA precipitates of PA-III cells showed that VLDL induced irreparable cellular damage during the initial 15 h of incubation.**

**The cytotoxic activity of VLDL was not due to the association with $PGF_{2\alpha}$, $\beta$-oestradiol, progesterone, 25-hydroxycholesterol, or free fatty acids (oleic, stearic, palmitic, linoleic, linolenic and arachidonic acids), monopalmitolein, elaidyl and $\alpha$-linolenyl alcohol.**

**The role of this factor in host defence against neoplasia is discussed.**

THE ASSOCIATION of serum lipoproteins with oncolytic activity was first reported in sera from women in advanced pregnancy against HeLa cells (Rejnek *et al.*, 1963). The concept that lipoproteins can function as containment factors in host defence against neoplasia was developed and substantiated with the observation that very-low-density lipoproteins (VLDL), isolated from sera of pregnant rats, manifest a wide spectrum of cytotoxicity against a number of tumour cell lines (Chan & Pollard, 1978*a*, *b*). Low-density lipoproteins (LDL) and high-density lipoproteins (HDL) had no cytotoxicity. The role of VLDL in host regulation of tumour metastasis was subsequently postulated, in the study in which heparin was shown to accelerate the spontaneous metastasis of implanted prostate adenocarcinoma cells (Chan & Pollard, 1980). The metastasis-enhancing effect of heparin was shown to be mediated *via* the release of lipoprotein lipases from the endothelial cells which act specifically on VLDL, thereby drastically reducing its cytotoxic titre.

We here report experiments that were designed to characterize further this VLDL-associated cytotoxic factor. These include (i) the subfractionation of VLDL by ultracentrifugal flotation, (ii) the delipidation of VLDL by n-heptane, (iii) chemical analysis of VLDL by thin-layer chromatography, and (iv) kinetic studies of inhibition of macromolecular

* To whom reprint requests should be addressed.

synthesis by VLDL. The possible role of VLDL as a surveillance agent in host defence against neoplasia is discussed.

### MATERIALS AND METHODS

*Animals and serum collection.*—The Lobund Wistar (LoW) rats (150–200 g), used throughout the study, have been maintained and propagated in our laboratory for 36 generations. Serum samples were collected from pregnant rats which were subjected to a timed-breeding schedule. At 36 h before parturition, rats were fasted for at least 18 h. They were then anaesthetized with ether and exsanguinated from the exposed heart. After storage at 4°C for 2 h, the serum was separated from the clot by centrifugation at 3000 *g* for 10 min. The serum was further clarified at 10,000 *g* for 10 min at 4°C. Lipoproteins were then isolated from the serum.

*Ultracentrifugal flotation.*—Total VLDL ($S_f$ 20–400) was isolated by preparative ultracentrifugation as described (Chan & Pollard, 1978*b*) at a density of 1·006. VLDL was subfractionated by the procedure of Gustafson *et al.* (1965), a type-40 rotor and a Beckman model L ultracentrifuge being used. Briefly, 2 ml of a buffer solution (consisting of 1·42 g anhydrous disodium phosphate, 7·27 g NaCl, and 0·1 g disodium EDTA in 1 l solution, at pH 7·0) of density 1·006 was layered carefully over 4 ml of serum in a Beckman polycarbonate centrifuge tube. The various VLDL subfractions ($S_f$ 20–50, 50–100, and 100–400) were separated by differential ultracentrifugation. The VLDL subfractions were washed once in buffer solution, and dialysed exhaustively against 0·15M NaCl containing 0·05% EDTA at pH 7·2 for 36 h. The dialysed VLDL was then flushed and stored in $N_2$ at 4°C. The VLDL was stable for 6–8 weeks without loss of oncolytic titre.

*Lipid analysis of VLDL.*—One ml of VLDL was extracted with 5 ml of chloroform–methanol (2:1, v/v) and the lipid extracts were washed with 0·2 volumes of 0·58% NaCl. The organic phase was evaporated under $N_2$ to a final volume of 0·5 ml, and was either stored at −20°C or used immediately for thin-layer chromatography and lipid quantitation.

Thin-layer chromatography (TLC) was carried out on 20 × 20 cm plates coated with silica gel G, 250 μm in thickness. The plates were pre-run in diethyl ether and then activated at 110°C for 30 min. After application of samples, plates were developed to 15 cm from the origin with diethyl ether-benzene-ethanol-acetic acid 40:50:2:0·2 (by volume), dried and transferred to another tank containing diethyl ether-hexane 6:94 and developed to within 1 cm of the top of the plate (Freeman & West, 1966). Lipid fractions were examined under UV light after spraying the plate with 0·05% Rhodamine 6G in 95% ethanol. Lipid classes were identified by comparison with the mobilities of known standards. The lipid standards were obtained from Supelco Co. (Bellefonte, Pennsylvania) and included cholesteryl oleate, tripalmitin, palmitic acid, cholesterol, and lecithin.

The lipid extracts were quantitated for triglycerides (Biggs *et al.*, 1975), total cholesterol (Allain *et al.*, 1974), and lipid phosphorus (Gindler & Ishizaki, 1969) with reagents from Sigma Co. (St Louis, Missouri). Calculation of phospholipid mass was made on the assumption that phosphorus accounts for 4% of the total phospholipid weight.

*Partial delipidation of VLDL.*—The neutral lipid core of VLDL was selectively extracted with n-heptane (Gustafson, 1965). Two ml of VLDL was lyophilized in the presence of 50 mg potato starch and extracted twice with 5 ml of redistilled n-heptane. The n-heptane extracts were pooled and evaporated under $N_2$ to a final volume of 1 ml for lipid analysis as described above. For the quantitation of oncolytic activity of the lipid extracts, the n-heptane phase was evaporated under $N_2$ to dryness, and reconstituted with 2 ml of Eagle's minimum essential medium (MEM) by sonication for 1 min. The reconstituted lipids were assayed for PA-III $CI_{50}$ endpoint (Chan & Pollard, 1978*b*). The final sediment, which consists of apoprotein–phospholipid complex, was dried under $N_2$ for 30 min at 4°C and extracted with 2 ml of 0·15M NaCl. The soluble residue was separated from the starch by low-speed centrifugation and assayed for oncolytic activity.

*Protein determination.*—Protein was determined by the method of Lowry, bovine serum albumin being used as a standard. Turbidity caused by high concentrations of lipids was resolved by the addition of 1 ml of 2% sodium deoxycholate before spectrophotometric reading.

*Tissue culture.*—The rat prostate adenocarcinoma cell lines (PA-III and PA-$III_f$)

(Chang & Pollard, 1977; Chan, 1981) were used as target cells to investigate the oncolytic activity of VLDL. Both lines have been cloned and reconstitute adenocarcinomas when inoculated into LoW rats, where they metastasize spontaneously *via* ipsilateral lymphatic channels to the lungs and produce lesions (Pollard & Luckert, 1979; Chan, 1981). The PA-III cells were propagated in MEM with 10% heat-inactivated foetal bovine serum (Grand Island Biological Co., N.Y.), plus penicillin 100 u/ml) and streptomycin (100 μg/ml). The PA-III$_f$ line is a subclone of PA-III, which has been adapted to grow in a serum-free medium supplemented with growth factors (Chan, 1981).

*Colony-inhibition assay.*—The colony-inhibiting assay was used to quantitate the cytotoxic activity of VLDL and its various subfractions. Both PA-III and PA-III$_f$ were used as target cells. The assay has been described previously (Chan & Pollard, 1978*b*). Serial dilutions of VLDL were made and the endpoint was defined as the reciprocal of the highest dilution of VLDL which would inhibit 50% of the colonies formed by the PA-III cells and expressed as colony inhibition ($CI_{50}$) units per ml of VLDL. The computation of PA-III $CI_{50}$ was calculated by the method of Reed & Muench (1938).

*DNA and protein synthesis.*—The effect of VLDL on the incorporation of radioactive thymidine and leucine into 10% trichloroacetic acid (TCA) precipitates of PA-III cells was studied as described (Chan, 1980). Leucine, L-($^{14}C$) (sp. act. 50 mCi/mmol) and thymidine (methyl-$^{3}H$, sp. act. 20 Ci/mmol) were from New England Nuclear (Boston, Massachusetts). The PA-III cells were pulse-labelled with radioactive precursor (1 μCi/ml) for 1 h at the end of the incubation with VLDL. The 10% TCA precipitates were collected on Millipore filters (HAWPO 2500) and counted in Biofluor, a Beckman LS 7000 liquid-scintillation counter being used.

### RESULTS

### *Lipid analysis of VLDL*

The 4 major lipid components of VLDL (triglycerides, esterified and free cholesterol, and phospholipids) were separated by thin-layer chromatography on plates coated with silica gel G, and were identified by matching distance of migration with lipid standards. Phospholipids remained at the origin of application, whilst esterified cholesterol moved the furthest from the origin, followed by triglycerides and then cholesterol as distinct bands. There was a drastic overall increase in the quantity of all the lipid constituents of VLDL from pregnant rats at 24 h before parturition when compared with the non-pregnant control (Table I). The increase in the triglyceride content was most pronounced—an almost 10-fold difference. There was no significant difference in the lipid content of VLDL from rats 24 h *post partum* or from non-pregnant rats.

### *Heptane delipidation of VLDL*

VLDL was lyophilized in the presence of potato starch and then extracted with n-heptane. The n-heptane extraction completely removed neutral lipids from VLDL, with preservation of a soluble apoprotein–phospholipid complex. As detected by TLC, a small portion of phospholipids was also removed during the heptane extrac-

TABLE I.—*Composition and oncolytic titre of VLDL* ($S_f$ 20–400) *isolated from sera of Lobund Wistar rats* (*LoW*)

| | VLDL constituents (mg/dl serum) | | | | PA-III§ |
|---|---|---|---|---|---|
| Rat | Protein | Triglyceride | Cholesterol | Phospholipid | $CI_{50}$ (u/ml VLDL) |
| LoW*g** | 30·5 ± 1·82‡ | 241·5 ± 10·86 | 11·25 ± 0·56 | 32·5 ± 1·4 | 252 ± 24 |
| LoW♀ | 6·1 ± 0·34 | 22·5 ± 1·19 | 1·50 ± 0·11 | 6·5 ± 0·37 | 35 ± 6 |
| $LoW_{pd}$† | 6·5 ± 0·33 | 23·75 ± 1·45 | 1·75 ± 0·12 | 6·75 ± 0·48 | 32 ± 5 |

* Pregnant females at 21 days of gestation.
† Rats at 24 h *post partum*.
‡ Mean ± s.d.
§ The PA-III $CI_{50}$ unit was defined as the reciprocal of the highest dilution of VLDL which would inhibit 50% of the colonies formed by PA-III cells.

TABLE II.—*Oncolytic activity of neutral lipids and phospholipid–protein residues obtained by heptane extraction of lyophilized VLDL ($S_f$ 20–400) isolated from sera of pregnant LoW rats*

| | Triglycerides (mg/ml) | Protein (mg/ml) | PA-III $CI_{50}$ (u/ml) |
|---|---|---|---|
| Neutral lipids* (heptane phase) | 9·15 ± 0·72 | 0·0086 ± 0·0012 | 204 ± 17 |
| Protein-phospholipid residue | 0 | 0·78 ± 0·08 | 0 |
| VLDL ($S_f$ 20–400) | 9·54 ± 0·87 | 1·134 ± 0·11 | 247 ± 23 |

* The heptane extracts were pooled and dried under $N_2$ and reduced pressure, and then reconstituted to 2 ml with MEM by sonication.

tion. As shown in Table II, oncolytic activity was detectable only in the reconstituted heptane phase, as assayed by colony inhibition. The soluble apoprotein–phospholipid residue had no cytotoxicity.

*Subfractionation of VLDL*

By means of sequential ultracentrifugal flotation, VLDL was fractionated into 3 subclasses according to their $S_f$ values: 20–50, 50–100, and 100–400. Each fraction was washed once to eliminate contamination, causing a 10% loss of mass and cytotoxicity (Table III). The $S_f$ 100–400 fraction contained most of the cytotoxicity.

*Effect of VLDL on growth of PA-III cells*

The effect of VLDL on the growth of PA-III cells was measured by the colony-inhibition assay, and the incorporation of radioactive thymidine and leucine into 10% TCA-precipitable materials.

VLDL was markedly cytotoxic to PA-III cells. Exposure to VLDL for 15 h was sufficient to kill all the target cells, even though the cultures were rinsed, fresh medium replenished, and the cells allowed to recover for 96 h. The inhibition of PA-III colony formation as a function of the hours of exposure to VLDL is shown in Fig. 1. There was a lag period of about 2 h, after which the % inhibited rose linearly and showed a plateau at 55% during 6–10 h exposure. The % inhibition rose again linearly to 100% cytotoxicity at 15 h.

The effects of VLDL on DNA and protein synthesis of PA-III cells were studied by pulse-labelling the cell cultures imme-

TABLE III.—*Oncolytic activity and neutral lipids of the* 3 *subfractions of VLDL* ($S_f$ 20–400) *isolated from sera of pregnant LoW rats*

| VLDL | Triglycerides (mg/dl serum) | Cholesterol (mg/dl serum) | PA-III $CI_{50}$ (u/ml) |
|---|---|---|---|
| $S_f$ 100–400* | 119·85 ± 9·55 | 8·34 ± 0·42 | 219 ± 19 |
| $S_f$ 50–100 | 32·0 ± 3·4 | 2·84 ± 0·11 | 21 ± 5 |
| $S_f$ 20–50 | 13·1 ± 1·55 | 0·75 ± 0·05 | 0 |
| $S_f$ 20–400 | 238·54 ± 16·78 | 12·45 ± 0·56 | 242 ± 23 |

* Each VLDL subfraction was isolated by sequential ultracentrifugal flotation at a density of 1·006 with "g. min" increased for each successive separation. $S_f$ 100–400 fraction was isolated at $4·8 \times 10^6$ g. min, $S_f$ 50–100 at $12·6 \times 10^6$ g. min, and $S_f$ 20–50 at $139 \times 10^6$ g. min.

FIG. 1.—Kinetics of PA-III cell colony inhibition following exposure to VLDL (25 μg protein/ml). The cells were rinsed and replenished with fresh medium at the end of exposure to VLDL and allowed to grow for another 96 h.

FIG. 2.—Time course of inhibition of radioactive thymidine and leucine incorporation into 10% TCA precipitates of PA-III cells by VLDL (25 μg protein/ml). The cells were pulse-labelled at the end of the incubation.

diately after the removal of VLDL. As shown in Fig. 2, the incorporation of [$^3$H]-dT and of $^3$H-leucine into 10% TCA precipitates was markedly suppressed during incubation. DNA synthesis was completely arrested in PA-III cells after 10 h of incubation with VLDL, and the decline in the rate of DNA synthesis appeared to be linear with time. However, there was no suppression of protein synthesis in the first 3 h of incubation, and then it proceeded linearly with time until the incorporation of $^3$H-leucine into protein was totally halted by the 12th hour of incubation.

*Effect of sterols and fatty acids on the growth of PA-III cells*

In order to determine whether the cytotoxicity of VLDL is due to the autooxidation of its cholesterol components into oxygenated sterols, the effect of 25-OH cholesterol on the proliferation of PA-III cells was examined. The PA-III cells were maintained in Waymouth 752/1 serum-free medium supplemented with insulin (5 μg/ml), transferrin (25 μg/ml), and fetuin (150 μg/ml). As shown in

TABLE IV.—*Inhibitory effect of VLDL and oxygenated sterols on DNA and protein synthesis of PA-III cells*

| Agents | Colony inhibition† (%) | % of inhibition of incorporation* $^3$H-thymidine | $^{14}$C-leucine |
|---|---|---|---|
| VLDL (50 μg/ml) | 100 | 96·7 | 93·4 |
| VLDL + Cholesterol (10 μg/ml) | 98·7 | 91·2 | 92·3 |
| 25-OH-Cholesterol (6 μg/ml) | 75·7 | 3·5 | 0 |
| 25-OH-Cholesterol + Cholesterol (6 μg/ml) | 0 | 0 | 0 |
| Cholesterol (10 μg/ml) | 0 | 0 | 0 |

* The incorporation of [$^3$H]-dT and $^{14}$C-leucine into TCA precipitates was measured after 24 h of incubation with the agents.

† The % of colony inhibition was scored at the end of 96 h incubation with the agents.

Table IV, 25-OH (5 μg/ml) showed pronounced inhibition of the proliferation of PA-III cells. 100% inhibition was found after 96 h of incubation. However, the inhibition of macromolecular synthesis was not apparent during the first 24 h of incubation. Both DNA and protein synthesis were suppressed significantly (80–90% inhibition of incorporation of radioactive precursors into 10% TCA precipitates) after 48 h of incubation with the sterol. The inhibitory effect of 25-OH can be reversed by adding cholesterol (5 μg/ml) to the medium. VLDL was extremely cytotoxic to the PA-III cells, as both DNA and protein synthesis were almost completely shut off at the end of 24 h incubation. The cytotoxicity of VLDL cannot be neutralized by the addition of cholesterol to the culture medium.

Other lipids which are known to be associated with VLDL were also tested for cytotoxicity against the PA-III cells by the colony-inhibition assay. Compounds tested include prostaglandin $F_{2\alpha}$, monopalmitolein, elaidyl and γ-linolenyl alcohol, β-oestradiol, progesterone and free fatty acids—oleic, stearic, palmitic, linoleic, linolenic, and arachidonic acid. The concentration of these lipids was 0·5–2·0 μg/

ml, and the assay was performed in serum-free conditions as described above. No cytotoxicity was observed in any of the above lipids, nor was the cytotoxicity of VLDL enhanced in the presence of these compounds.

### DISCUSSION

The role of lipoproteins in the transport of triglycerides and cholesterols for tissue metabolism and their ability to regulate the biosynthesis of cellular cholesterol *via* specific receptors have been recognized and documented (Goldstein & Brown, 1974, 1977). Recently new biological roles of lipoproteins have been reported. These include: (i) neutralization of xenotropic C-type virus (Leong *et al.*, 1977; Kane *et al.*, 1979); (ii) inhibition of the haemagglutination of Rubella virus (Shortridge & Ho, 1974; Steinman, 1976); (iii) regulation of mitogenic response of lymphocytes (Curtiss & Edgington, 1976, 1977; Hui & Harmony, 1980); (iv) modulation of the tumoricidal activity of macrophages (Chapman & Hibbs, 1977); (v) lysis of trypanosomes (Rifkin, 1978); (vi) inhibition of granulopoiesis in marrow cultures (Mortensen *et al.*, 1979); and (vii) inhibition of colony formation by prostate adenocarcinoma cells (Chan & Pollard, 1978*a*, *b*). The oncolytic activity of VLDL and its regulatory role in tumour metastasis have been postulated (Chan & Pollard, 1978*b*, 1980). Our present report aims at a better understanding of the chemical nature of this VLDL-associated cytotoxicity and its interaction with prostate adenocarcinoma cells.

The chemical analysis of VLDL isolated from sera of pregnant rats 24 h before and after parturition (Table I) indicates that high levels of VLDL had accumulated in serum at advanced gestation. This is in accordance with the report that pregnant women had very high values for total VLDL during late pregnancy (Barclay, 1972). The values for VLDL components in the sera of pregnant rats showed an 8–9-fold increase over those in virgin rats. The most pronounced increase was due to neutral lipids. There was also a concomitant 8-fold increase in the cytotoxic titre of VLDL in the sera from pregnant rats. The level of VLDL declined to normal at 24 h after parturition. This confirms our previous observation that the cytotoxicity of sera from pregnant rats peaks before parturition and disappears thereafter (Chan & Pollard, 1978*b*). The physiological significance of serum hyperlipaemia before parturition remains obscure. One possible interpretation is the transfer of arachidonic acid from plasma lipoproteins to luteal tissues for prostaglandin synthesis. A 10-fold rise in lutein triglyceride concentrations has been reported in pregnant rats 48 h before parturition (Strauss *et al.*, 1977). It has been suggested that prostaglandins produced within the lutein cells might induce the regression of corpus luteum (Strauss *et al.*, 1977). The increase in cytotoxicity of VLDL during late pregnancy could function as a defence of the mother against the influx of allogeneic cells from the foetuses. The existence of such non-immunological host defence has been proposed (Apffel, 1976; Prehn, 1977). Our observation on the cytotoxicity of VLDL tends to support such a postulate.

The identification of the component of VLDL that confers cytotoxicity is unresolved. We have tested a number of lipids known to be associated with VLDL, which include saturated and unsaturated free fatty acids, unsaturated monoglycerides and alcohols, steroid hormones and prostaglandins. Yet, at physiological concentrations, none of these compounds have any cytotoxic effects on the PA-III cells. The lyophilization of VLDL in the presence of potato starch preserved the phospholipid–apoprotein complex in soluble form, and prevented aggregation during heptane extraction. The recovery of cytotoxicity in the reconstituted heptane extracts, and the failure of the apoprotein–phospholipid complex to demonstrate any cytotoxicity against the PA-III cells, suggest that the neutral lipids of VLDL are the cytotoxic components. However, the identity of these components has yet to be determined.

One possible explanation for the observed cytotoxicity of VLDL is the autooxidation of its cholesterol into oxygenated derivatives such as 25-hydroxycholesterol (25-OH). These oxygenated sterols have been reported to be potent inhibitors of *de novo* cholesterol biosynthesis in tissue culture cells, with subsequent cell death (Kandutsch & Chan, 1977; Kandutsch *et al.*, 1978). However, such an interpretation cannot be supported, since the supplementation of culture medium with cholesterol did not reverse the cytotoxicity of VLDL, whilst the inhibitory effect of 25-OH on the colony formation of PA-III cells can be neutralized by cholesterol. Furthermore, kinetic studies (Table IV) indicate that VLDL was able to arrest DNA and protein synthesis of PA-III cells within 12 h of treatment, whereas 25-OH required more than 36 h to exert its inhibition effects. Although the kinetic studies of the inhibition of macromolecular synthesis (Figs. 1 and 2) and colony formation of PA-III cells by VLDL has provided some understanding of the time course of cellular events after exposure to VLDL, the actual mechanism of oncolysis remains unknown.

By means of differential ultracentrifugal flotation, VLDL was separated into 3 subfractions, the $S_f$ 100–400 fraction conferring most of the cytotoxicity (Table III). It has been reported that cancer patients with various types of malignancies, such as melanoma, leukaemia and lymphoma, had high values for total VLDL, and the $S_f$ 100–400 fraction was very high (Barclay & Skipski, 1975). Similar observations were made in rats bearing Walker carcinosarcoma 256, and the neutral lipids of VLDL, especially the triglycerides, were significantly high (Barclay *et al.*, 1962, 1967). We have also observed that rats developing primary colon tumours and a high level of VLDL, accompanied by a significant increase in the cytotoxic titre of VLDL (unpublished) It thus appeared that the VLDL-mediated surveillance mechanisms, as suggested above, are operative *in vivo*, and deserve further investigation.

This work was supported in part by US PHS Grant CA-17559 and a grant from the Cancer Society of St Joseph County, Indiana. We thank Vicky Rodebush for excellent secretarial assistance.

### REFERENCES

ALLAIN, C. C., POON, L. S., CHAN, C. S. G., RICHMOND, W. & FU, P. C. (1974) Enzymatic determination of total serum cholesterol. *Clin. Chem.*, **20**, 470.

APFFEL, C. A. (1976) Nonimmunological host defenses: A Review. *Cancer Res.*, **36**, 1527.

BARCLAY, M., CALATHES, D. N., GARFINKEL, E., TEREBUS-KEKISH, O., BARCLAY, R. K. & SKIPSKI, V. P. (1962) Low-density lipoproteins and lipoprotein lipase activity in tissues from rats bearing Walker carcinosarcoma 256. *Arch. Biochem. Biophys.*, **98**, 391.

BARCLAY, M., SKIPSKI, V. P., TEREBUS-KEKISH, O., MERKER, P. L. & CAPPCUCINO, J. G. (1967) Serum lipoproteins in rats with tumors induced by 9,10-dimethyl-1,2-benzanthracene and with transplanted Walker carcinosarcoma 256. *Cancer Res.*, **27**, 1158.

BARCLAY, M. & SKIPSKI, V. P. (1975) Lipoproteins in relation to cancer. *Progr. Biochem. Pharmacol.*, **10**, 76.

BARCLAY, M. (1972) Lipoprotein class distribution in normal and diseased states. In *Blood Lipids and Lipoproteins: Quantatition, Composition, and Metabolism*. Ed. Nelson. New York: John Wiley. p. 585.

BIGGS, H. G., ERIKSON, J. M. & MOOREHEAD, W. R. (1975) A manual colorimetric assay of triglycerides in serum. *Clin. Chem.*, **21**, 437.

CHAN, S. Y. (1980) Androgen and glucorticoid receptors in the Pollard prostate adenocarcinoma cell lines. *The Prostate*, **1**, 53.

CHAN, S. Y. (1981) A chemically-defined medium for the propagation of prostate adenocarcinoma cells. *The Prostate*, **2** (in press).

CHAN, S. Y. & POLLARD, M. (1978*a*) Oncolytic factor(s) in serum of pregnant rats. *Fed. Proc.*, **37**, 161.

CHAN, S. Y. & POLLARD, M. (1978*b*) *In vitro* effects of lipoprotein-associated cytotoxic factor on rat prostate adenocarcinoma cells. *Cancer Res.*, **38**, 2956.

CHAN, S. Y. & POLLARD, M. (1980) Metastasis-enhancing effect of heparin and its relationship to a lipoprotein factor. *J. Natl Cancer Inst.*, **64**, 1121.

CHANG, F. C. & POLLARD, M. (1977) *In vitro* propagation of prostate adenocarcinoma cells from rats. *Invest. Urol.*, **14**, 331.

CHAPMAN, H. A., JR & HIBBS, J. B. (1977) Modulation of macrophage tumoricidal capability by components of normal serum: A central role for lipid. *Science*, **197**, 282.

CURTISS, L. K. & EDGINGTON, T. S. (1976) Regulatory serum lipoproteins: Regulation of lymphocyte stimulation by a species of low density lipoprotein. *J. Immunol.*, **116**, 1452.

CURTISS, L. K. & EDGINGTON, T. S. (1977) Effect of

LDL-In, a normal immunoregulatory human serum low density lipoprotein, on the interaction of macrophages with lymphocytes proliferating in response to mitogen and allogeneic stimulation. *J. Immunol.*, **118**, 1966.

FREEMAN, C. P. & WEST, D. (1966) Complete separation of lipid classes on a single thin-layer plate. *J. Lipid Res.*, **7**, 324.

GINDLER, E. M. & ISHIZAKI, R. T. (1969) Rapid semimicro colorimetric determination of phosphorus in serum and nonionic surfactants. *Clin. Chem.*, **15**, 807.

GOLDSTEIN, J. L. & BROWN, M. S. (1974) Binding and degradation of low density lipoproteins by cultured human fibroblasts. *J. Biol. Chem.*, **249**, 5153.

GOLDSTEIN, J. L. & BROWN, U. M. (1977) Atherosclerosis: The low-density lipoprotein receptor hypothesis. *Metabolism*, **26**, 1257.

GUSTAFSON, A., ALAUPOVIC, P. & FURMAN, R. H. (1965) Studies of the composition and structure of serum lipoproteins: Isolation, purification, and characterization of very low density lipoproteins of human serum. *Biochemistry*, **4**, 596.

GUSTAFSON, A. (1965) New method for partial delipidization of serum lipoproteins. *J. Lipid Res.*, **6**, 512.

HUI, D. Y. & HARMONY, J. A. K. (1980) Immunoregulatory plasma lipoproteins. *J. Biol. Chem.*, **255**, 11775.

KANDUTSCH, A. A. & CHEN, H. W. (1977) Consequences of blocked sterol synthesis in cultured cells. *J. Biol. Chem.*, **252**, 409.

KANDUTSCH, A. A., CHEN, H. W. & HEINIGER, H. (1978) Biological activity of some oxygenated sterols. *Science*, **201**, 498.

KANE, J. P., HARDMAN, D. A., DIMPFL, J. C. & LEVY, J. A. (1979) Apolipoprotein is responsible for neutralization of xenotropic type C virus by mouse serum. *Proc. Natl Acad. Sci. U.S.A.*, **76**, 5957.

LEONG, J. C., KANE, J. P., OLESZKO, O. & LEVY, J. A. (1977) Antigen-specific non-immunoglobulin factor that neutralizes xenotropic virus is associated with mouse serum lipoproteins. *Proc. Natl Acad. Sci. U.S.A.*, **74**, 276.

MORTENSEN, B. T., KNUDTZON, S. & SCHULTZ, H. (1979) Serum lipoprotein inhibitors of granulopoiesis in human bone marrow cultures. *Exp. Hematol.*, **7**, 1.

POLLARD, M. & LUCKERT, P. (1979) Patterns of spontaneous metastases manifested by three rat prostate adenocarcinomas. *J. Surg. Oncol.*, **12**, 371.

PREHN, R. T. (1977) Immunostimulation of the lymphodependent phase of neoplastic growth. *J. Natl Cancer Inst.*, **59**, 1043.

REID, L. J. & MUENCH, H. (1938) A simple method of estimating fifty per cent end points *Am. J. Hyg.*, **27**, 493.

REJNEK, J., BEDNARIK, T., RERABKOVA, E. & DOLEZAL, A. (1963) Investigations of the influence of sera from pregnant women on the growth of cell cultures in conjunction with the occurrence of abnormal $\alpha_1$-lipoprotein. *Clin. Chim. Acta*, **8**, 108.

RIFKIN, M. R. (1978) Identification of the trypanocidal factor in normal human serum: High density lipoprotein. *Proc. Natl Acad. Sci. U.S.A.*, **75**, 3450.

SHORTRIDGE, K. F. & HO, W. K. K. (1974) Human serum lipoproteins as inhibitors of hemagglutination for selected Togaviruses. *J. Gen. Virol.*, **23**, 113.

STEINMANN, J. (1976) Lipoproteins as serum inhibitors in Rubella hemagglutination inhibition test and their elimination. *Zentralbl. Bakteriol.*, **239**, 10.

STRAUSS, J. F., III, SEIFTER, E., LIEN, E. L., GOODMAN, D. B. P. & STAMBAUGH, R. L. (1977) Lipid metabolism in regressing rat corpora lutea of pregnancy. *J. Lipid Res.*, **18**, 246.